# Sealing ability, marginal adaptation and their correlation using three root-end filling materials as apical plugs

**DOI:** 10.1590/S1678-77572010000200006

**Published:** 2010

**Authors:** Fernando Accorsi OROSCO, Clovis Monteiro BRAMANTE, Roberto Brandão GARCIA, Norberti BERNARDINELI, Ivaldo Gomes de MORAES

**Affiliations:** 1 DDS, MSc, PhD student in Endodontics, Department of Operative Dentistry, Endodontics and Dental Materials, Bauru School of Dentistry, University of São Paulo, Bauru, SP, Brazil.; 2 DDS, MSc, PhD, Full Professor of Endodontics, Department of Operative Dentistry, Endodontics and Dental Materials, Bauru School of Dentistry, University of São Paulo, Bauru, SP, Brazil.; 3 DDS, MSc, PhD, Associate Professor of Endodontics, Department of Operative Dentistry, Endodontics and Dental Materials, Bauru School of Dentistry, University of São Paulo, Bauru, SP, Brazil.

**Keywords:** Sealing ability, Marginal adaptation, Root-end filling materials

## Abstract

**Objective:**

This study used dye leakage assay and scanning electron microscopy to evaluate, respectively, the sealing ability and marginal adaptation of three root-end filling materials used as apical plugs, as well as the possible correlation between these properties.

**Material and Methods:**

Ninety-eight single-rooted human teeth were prepared to simulate an open apex. The teeth were allocated to three experimental groups (n = 30), which received a 5-mm thick apical plug of (1) gray MTA Angelus^TM^, (2) CPM^TM^ and (3) MBPc, and two controls groups (n = 4). After immersion in 0.2% Rhodamine B solution for 48 h, the teeth were sectioned longitudinally and analyzed by Image Tool 3.0 software. The marginal adaptation between apical plugs and the root canal walls were analyzed by SEM.

**Results:**

MBPc had significantly less (p<0.05) apical leakage than the other materials. Regarding marginal adaptation, CPM^TM^ showed the best numerical results, though without statistical significance from the other materials (p<0.05). There was no correlation between the two properties.

**Conclusions:**

When used as apical plugs, the tested root-end filling materials had similar marginal adaptation to the dentin walls, but MBPc had the best sealing ability, as demonstrated by the least apical leakage from all tested materials.

## INTRODUCTION

When immature teeth develop pulp necrosis, dentin formation is interrupted and root development ceases. Consequently, the root canal is large, with thin and fragile walls, and the apex remains open^10^. The aim of the treatment of teeth with open apex is to seal a sizeable communication between the root canal system and the periradicular tissue, and provide a barrier against which filling material can be compacted^3^. Materials such as calcium hydroxide and, more recently, mineral trioxide aggregate (MTA), have been used as apical plugs^10^.

MTA was developed by Torabinejad in the early 1990s, and the first study on this material was published by Lee, et al.^18^ (1993). In 2001, a Brazilian company (Angelus Soluções Odontológicas, Londrina, PR, Brazil) introduced to the market the MTA developed in Brazil, which is apparently identical to the MTA developed by Torabinejad^9,15^. The main components of MTA are tricalcium oxide, tricalcium silicate, bismuth oxide, tricalcium aluminate, tricalcium oxide, tetracalcium aluminoferrite and silicate oxide. In addition, there are a few other mineral oxides, which are responsible for the chemical and physical properties of MTA. The powder consists of fine hydrophilic particles that form a colloidal gel in the presence of water or moisture; this gel solidifies to form a hard sealer in less than 4h^31^.

In 2004, a material similar to MTA was developed for clinical use in Argentina under the brand name CPM^TM^ (Egeo S.R.L., Buenos Aires, Argentina). The powder also consists of fine hydrophilic particles that form a colloidal gel in presence of moisture, solidifying to form a hard sealer in 1 h. The main components are tricalcium silicate, tricalcium oxide, tricalcium aluminate and other oxides^6^.

In 1984, the investigators Moraes and Berbert, from the Department of Operative Dentistry, Endodontics and Dental Materials at Bauru Dental School, University of São Paulo, Brazil., developed a epoxy resin sealer containing calcium hydroxide (MBPc), which was introduced as a root-end filling material. MBPc is packed in glass vials as a hydrophobic paste/paste sealer, mixed in a 4:1 ratio (base paste:catalyst paste), with 4 h of setting time^7^.

The quality of apical sealing obtained by rootend filling materials has been assessed using different methodologies such as dye penetration^5,16,18,23,28,29,33^, bacterial penetration^3,14^, endotoxin^26^, human saliva penetration^2^ and fluid filtration technique^17,21^. Studies on dye penetration were considered an easy method to evaluate root-end filling materials^4,33^. Scanning electron microscopy (SEM) has also been used to assess the adaptation and the sealing ability of root-end filling materials^1,13,25,33^.

The purposes of this study were to evaluate the sealing ability, by dye leakage, and the marginal adaptation, by SEM, of apical plugs fabricated with gray MTA Angelus^TM^, CPM^TM^ and MBPc, as well as to verify the existence of a correlation between apical leakage and marginal adaptation in the tested materials.

## MATERIAL AND METHODS

### Specimen Preparation

The study was approved by the Institutional Review Board of Bauru Dental School (133/2005). Ninety-eight extracted single-rooted human teeth (upper central and lateral incisors) were used for this study. The teeth were stored in 10% formalin for a period of 8 weeks^11^ and kept moist before the experiment. The tooth crowns were removed below the cementoenamel junction to obtain a standard root length of 13 mm.

### Canal Preparation

At first, the canals were instrumented with Gates Glidden burs #5 up to #1 (Dentsply-Maillefer Instruments SA, Ballaigues, Switzerland) according to the crown-down technique until the #1 size bur could pass through the apical foramen. The specimens were then prepared with K files (Dentsply-Maillefer Instruments SA, Ballaigues, Switzerland), starting with an ISO file #50 until an ISO file #90 could be visualized 1 mm beyond the apex. The root canals were irrigated with 1mL of 1% sodium hypochlorite (Biodinâmica Química e Farmacêutica Ltda., Ibiporã, PR, Brazil) throughout instrumentation. The canals were filled with 1 mL of 17% EDTA (Biodinâmica Química e Farmacêutica Ltda) for 5 min and then dried with paper points (Tanariman Industrial Ltda, Manacapuru, AM, Brazil).

The teeth were then allocated to 3 experimental groups (n=30), according to the sealer used for fabrication of the 5-mm-thick apical plug - gray MTA Angelus^TM^, CPM^TM^ and MBPc, and 2 control groups (n=4), which did not receive an apical plug. In the experimental groups, the external surface of each root, except for the apical foramen, was made impermeable by application of a layer of epoxy adhesive (Araldite-Ciba-Geigy, Taboão da Serra, SP, Brazil), followed by two coats of nail polish (Cosbra Cosméticos Ltda., São Paulo, SP, Brazil)^8,24^. In the negative controls, the external surface of each root, including the apical foramen, was made impermeable^8,24^; in the positive controls, the external surface of each root was made impermeable, except for the apical foramen^8,24^.

### Apical Plugs

Gray MTA Angelus^TM^ was prepared following the manufacturer’s instructions, mixed at a 1:1 ratio (powder:sterile water) and carried with a Lentulo spiral (Dentsply-Maillefer Instruments SA, Ballaigues, Switzerland) at low speed up to 3 mm short of the apical foramen. The MTA was condensed up to the apical end with an ISO K file #90 wrapped in cotton. Another K file involved with moistened cotton was used to remove the excess MTA from the dentin walls. In case of overfilling, the excess material was also removed.

CPM^TM^ was also prepared according to the manufacturer’s instructions, mixed at a 3:1 ratio (powder:saline solution) and carried with a Lentulo spiral at low speed, in the same way as described for gray MTA Angelus^TM^. CPM^TM^ condensation and excess removal was performed as described for the MTA.

MBPc was mixed at a 4:1 ratio (base paste:catalyst paste). Before mixture, small cylindrical portions of the sealer were prepared, with smaller diameter than the root canal diameter. These cylinders were individually placed in the root canal using an ISO K file #70 up to the root canal end. The MBPc was condensed with pluggers and any overfilling material was removed with care avoid compressing the sealer against the apex. Radiographs were obtained from all teeth to check the thickness of the apical plug.

After fabrication of apical plugs, the remaining root canal portions were filled with a calcium hydroxide water-based paste (Odontopharma Indústria e Comércio Ltda, Porto Alegre, RS, Brazil) and placed in an oven at 37ºC for 15 days. After this period, the calcium hydroxide waterbased paste was removed by irrigation with saline, with aid an ISO K file #100. The root canals were dried with paper points (Tanariman Industrial Ltda, Manacapuru, AM, Brazil) and filled by the lateral condensation technique., using gutta-percha points (Tanariman Industrial Ltda, Manacapuru, AM, Brazil) and endodontic sealer (Sealer 26) (Dentsply Indústria e Comércio Ltda, Petrópolis, RJ, Brazil).

### Apical Leakage

The root surfaces were isolated with one layer of Araldite and two layers of nail polish. The cervical portion of each root was recovered by immersion in sticky wax followed by application of two layers of nail polish. The teeth of each group, properly identified, were fixated on utility wax and were placed in plastic flasks, leaving the apex free and facing upwards. The flasks were filled with 0.2% Rhodamine B solution (Labsynth Produtos para Laboratórios Ltda, Araçatuba, SP, Brazil; pH 7.0) in such a way to completely cover the apex of all teeth. The flasks were kept at 37ºC for 48 h. After that period, the teeth were removed from the dye, washed in running tap water for 24 h, dried and sectioned longitudinally.

Then, sets of five specimens were placed on a sheet of utility wax and photographed with a digital camera (Canon EOS Rebel 300 D) fixed on a tripod. The teeth were also photographed close to a millimeter plastic ruler. For analysis of the sealing ability of the tested materials, leakage of 0.2% Rhodamine B was linearly measured on the photographs using the software Image Tool 3.0. Leakage measurement considered the line with longer length of dye, on the apical plugdentin wall interface, from the most apical to the most cervical portion. Statistical analysis of the results was performed using the Kruskal-Wallis and the Dunn tests at 5% significance level.

### Marginal Adaptation

The marginal adaptation was evaluated in the 90 specimens (halves). These segments were gold-sputtered and analyzed by JEOL JSM T-220 (JEOL, Tokyo, Japan) SEM at ×35 and ×150 magnifications. For analysis of the marginal adaptation of the root-end filling materials, the photomicrographs at ×35 magnification were analyzed on the software Image Tool 3.0 and the extent of gap was measured linearly, in micrometers. Statistical analysis of the results was performed using the Kruskal-Wallis and the Dunn tests at 5% significance level.

## RESULTS

The sealing ability of the apical plugs fabricated from the different root-end filling materials can be classified, in descending numerical order of apical leakage, as follows: MBPc (1.99 ± 1.44 mm), Gray MTA-Angelus^TM^ (3.39 ± 1.39 mm) and CPM^TM^ (4.00 ± 1.00 mm). MBPc had significantly less (p<0.05) apical leakage than the other materials (Table 1).

**Table 1:** Comparison of mean dye leakage (mm) and standard deviation (SD) of root-end filling materials used as apical plugs

**Materials**	**Number of teeth**	**Mean ± SD (mm)**
Gray MTA Angelus™	30	3.39 ± 1.39ª
CPM™	30	4.00 ± 1.00ª
MBPc	30	1.99 ± 1.44^b^

**Figure 1:**
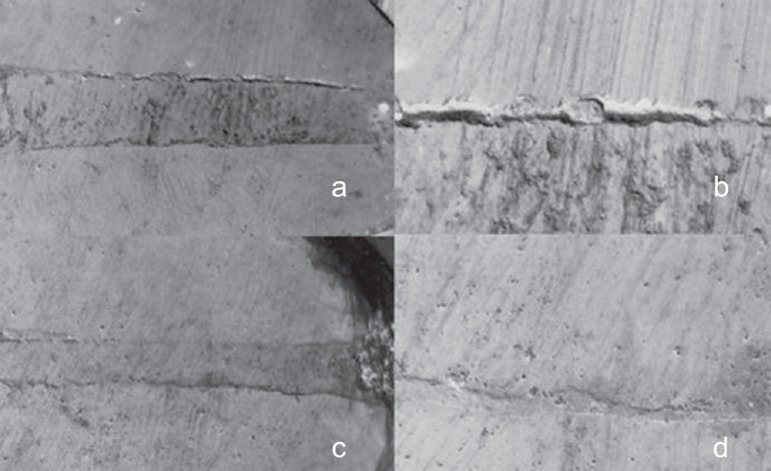
Panel of SEM micrographs of a tooth retrofilled with MTA-Angelus^TM^. a - Gray MTA-Angelus^TM^ apical plug (original magnification ×35); b - Gaps between gray MTA-Angelus^TM^ and dentin (original magnification ×150); c - Gray MTA-Angelus^TM^ apical plug (original magnification ×35); d - Marginal adaptation between gray MTA-Angelus^TM^ and dentin (original magnification ×150)

**Figure 2:**
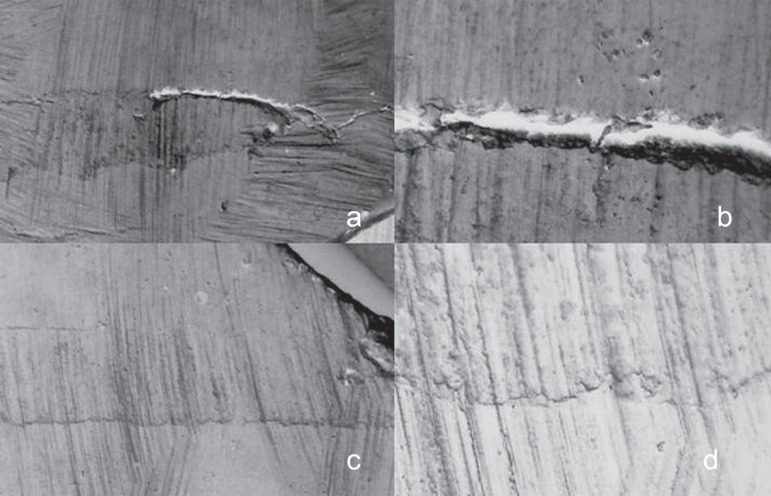
Panel of SEM micrographs of a tooth retrofilled with CPM^TM^. a -CPM^TM^ apical plug (original magnification ×35); b - Gaps between CPM^TM^ and dentin (original magnification ×150); c - CPM^TM^ apical plug (original magnification ×35); d - Marginal adaptation between CPM^TM^ and dentin (original magnification ×150)

**Figure 3:**
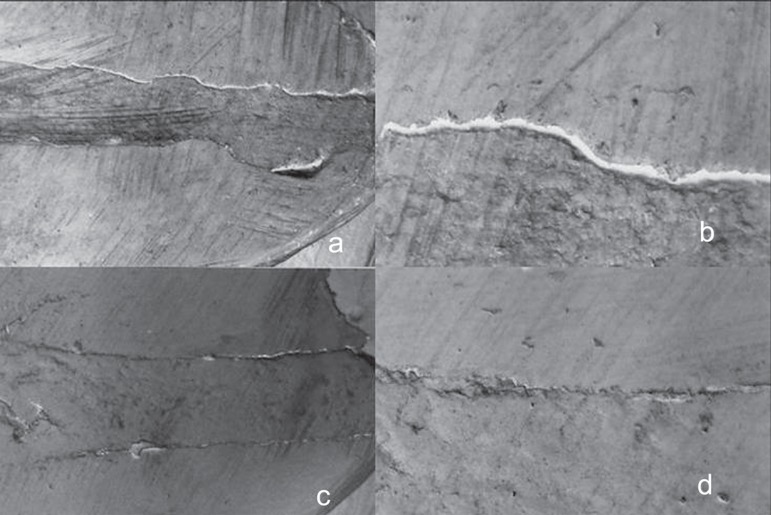
A - Panel of SEM micrographs of a tooth retrofilled with MBPc. a - MBPc apical plug (original magnification ×35); b - Gaps between MBPc and dentin (original magnification ×150); c - MBPc apical plug (original magnification ×35); d - Marginal adaptation between MBPc and dentin (original magnification ×150)

SEM examination of the specimens showed multiple gaps between apical plugs and dentin walls (Figures 1-3). The marginal adaptation of the apical plugs fabricated from the different rootend filling materials can be classified, in descending numerical order of marginal gap size, as follows: CPM^TM^ (337.71 ± 561.93µm), gray MTA-Angelus^TM^ (395.21 ± 760.58µm) and MBPc (474.11 ± 872.13µm). CPM^TM^ presented the smallest gaps in extension, but there no statistically significant difference (p>0.05) among the root-end filling materials regarding gap size (Table 2).

**Table 2:** Comparison of mean gap (pm) and standard deviation (SD) of root-end filling mateials used as apical plugs

**Materials**	**Number of teeth**	**Mean ± SD (mm)**
Gray MTA Angelus™	30	395.21 ± 760.58
CPM™	30	337.71 ± 561.93
MBPc	30	474.11 ± 872.13

The absence of correlation between the two properties was clearly observed by the Spearman’s Linear Correlation (p>0.05). This could be explained because the T values calculated for the apical plugs and their interaction according to Spearman’s Linear Correlation Coefficient were lower than the T_0.05_, showing the absence of correlation between apical leakage and marginal adaptation in this study.

## DISCUSSION

In the present study, dye penetrated the entire length of the positive controls, and there was no dye penetration in negative controls.

Several methodologies can be used to evaluate apical leakage. Among these we could mention endotoxin^25^, human saliva^2^, fluid filtration^17,21^ and bacterial leakage tests^3,14^. However, we agree with Aqrawabi^4^ who stated that if root-end filling materials were able to prevent leakage of small particles such as dye, they would possibly prevent the penetration of bacteria and their subproducts.

Various substances have been used to delineate apical leakage. Among the dyes, the use of methylene blue at different concentrations is outstanding^18,22,29^. However, Wu, et al.^32^ (1998) conducted an interesting work and stated that methylene blue suffers discoloration when in contact with some alkaline filling materials, which may cause unrealistic results of such materials in leakage studies. Methylene blue discoloration occurs because it is unstable when in contact with alkaline materials. Such materials cause hydrolysis of methylene blue, resulting in formation of a clear compound named thionine. This would explain why methylene blue is discolored by calcium hydroxide. In relation to MTA, in the presence of water, calcium oxide in the material could form calcium hydroxide, which would certainly cause discoloration of methylene blue. In this study, a 0.2% Rhodamine B solution was used because according to Moraes, et al.^23^ (2005) and Tanomaru Filho, et al.^27^ (2005) , Rhodamine B is not influenced by alkaline materials.

Regarding marginal adaptation analysis, Torabinejad, et al.^29^ showed that the specimen preparation for SEM investigation could create artificial/artifacts gaps within the interface. The authors suggest the use of resin replicas to avoid artificial gaps. At first, we tried to use this methodology in our study, but the resin replicas obtained were not sufficient good. So, considering other studies^24,33^ that showed satisfactory results using gold-sputtering and SEM investigation we adopted this methodology to evaluate the marginal adaptation. All specimens were evaluated because no artificial/artifacts gaps within the interface dentin wall/root-end filling material occurred.

Investigation of gray MTA was based on the frequency of utilization when apical plugs are necessary, with excellent results^12,20^. Gray MTA-Angelus^TM^ was used instead of gray ProRoot MTA^TM^ (Dentsply/Tulsa Dental, USA) in order to use a national product, which is also easier to find in the market.

According to the manufacturer, CPM^TM^ has similar or better physical, chemical and biological characteristics compared to MTA, with the same clinical indications^6^. As this material is also a mineral trioxide aggregate, this study evaluated the possibility of using it as apical plug, as well as its sealing ability and marginal adaptation, since few studies are available on CPM^TM^.

MBPc was also used because its physical and chemical characteristics have been assessed, showing great results^24^. A previous study has shown similar biological response to that of ProRoot MTA^7^. Since the initial clinical indications of this material included only use for root-end filling and filling of root perforation^24^, the possibility of using this material for fabrication of apical plug was investigated in the present study.

Calcium hydroxide was used before the root canal filling to allow the materials to set. A research study^22^ and some case reports^12,20^ support the two-step technique over the onestep procedure. Besides, calcium hydroxide, when used as an intracanal medication, should stay in the root canal, at least, for 15 days^19^.

The study of Bramante, et al.^6^ (2006) allows comparison between CPM^TM^ and the present results. According to the authors, CPM^TM^ has dimensional adhesion stability through time, among other properties. However, the results observed for this material with regard to sealing ability were not so good, with a mean overall leakage of 4.00 ± 1.00 mm.

With regard to MTA, the great sealing ability of both Pro Root^TM^ MTA and gray MTA-Angelus^TM^, used in this study, were highlighted by several authors^18,22,29,33^. Conversely, in the study of Silva Neto and Moraes^24^, MTA was not considered a good sealer. When used as apical plug, especially with 4- to 5-mm thickness, MTA has shown great sealing ability^3,14,22,31^. Consequently, the results observed for gray MTA-Angelus^TM^ in this study confirm those found in the aforementioned studies.

MBPc has been shown to have good sealing ability as a root-end filling material^24^. Similar behavior has been observed in the present study, in which MBPc had the best results with only 1.99 ± 1.44 mm of leakage, with statistically significant difference in relation to CPM^TM^ and gray MTA-Angelus^TM^.

Regarding the two variables analyzed in this study, there was clearly a lack of correlation between apical leakage rates and gaps at the dentin wall/root-end filling material interface. These findings are contrary to those of Stabholz, et al.^25^ (1985) ,who evaluated, *in vitro*, the marginal adaptation of retrograde fillings with Restodent, zinc phosphate cement, Cavit-W, Duralon and amalgam by SEM. The results were compared with those of a previous *in vitro* study which used a radionuclidic model for comparing sealability of the same five retrograde fillings. A correlation was established between marginal adaptation and sealing ability. The results demonstrated that Restodent showed the best marginal adaptation as well as its sealability was significant superior to that of the other four materials. Amalgam showed the poorest marginal adaptation and sealability. However, our results were similar to those found by Abdal and Retief^1^ (1982) and Xavier, et al.^33^ (2005), which also reported lack of correlation between apical leakage and marginal adaptation of the materials. In the Abdal and Retief^1^ (1982) study the *in vitro* apical seal obtained by post resection filling with heat-sealed gutta-percha alone and when reinforced with 16 retrofilling materials was evaluated qualitatively by SEM and quantitatively by a dye penetration technique. The results indicated that heat-sealed gutta-percha alone and when reinforced with a composite dental resin (Adaptic) or a glass ionomer cement (ASPA) provided the most effective apical seal. Xavier, et al.^33^ (2005) evaluated the root-end sealing ability through dye leakage evaluation and the marginal adaptation through SEM of MTA-Angelus, Super EBA and Vitremer. Concerning marginal adaptation, MTA-Angelus presented the best results, while Super EBA showed superior results when considering sealing ability. In our study, CPM^TM^ was the best root-end filling material considering marginal adaptation. Nevertheless, when analyzed the apical leakage, CPM^TM^ presented the worst results.

Regarding the possible correlation between sealing ability and marginal adaptation in this study, it is very important consider the limitations of the methodology used Although sealing ability and marginal adaptation were evaluated in the same points, it is impossible to affirm that what the SEM shows in that point occurred in all canal perimeters. Perhaps marginal adaptations can occurred in some points, even if where SEM showed gaps at the dentin wall/root-end filling material interface. If we considerer sealing ability as the seal reproduction in the whole canal perimeter, a gap maybe not represent what really occurred in the entire apical plug. Therefore, it is really hard to establish a correlation between sealing ability and marginal adaptation.

## CONCLUSIONS

When used as apical plugs, the tested rootend filling materials had similar marginal adaptation to the dentin walls, but MBPc had the best sealing ability, as demonstrated by the least apical leakage from all tested materials. It is possible to observe that the lack of gaps at the interface between the root-end filling material and the dentin walls did not hinder dye penetration.
